# A systematic review of the prevalence of germline pathogenic variants in patients with pancreatic cancer

**DOI:** 10.1007/s00535-021-01806-y

**Published:** 2021-07-13

**Authors:** Esteban Astiazaran-Symonds, Alisa M Goldstein

**Affiliations:** 1 Division of Cancer Epidemiology and Genetics, NCI, NIH, Rockville, MD, USA; 2 National Human Genome Research Institute, NIH, Bethesda, MD, USA

**Keywords:** Pancreatic cancer, pancreatic ductal adenocarcinoma, pathogenic variant, prevalence, mutation

## Abstract

The genetics of pancreatic ductal adenocarcinoma (PDAC) is complex with patients reported to harbor germline pathogenic variants (PVs) in many different genes. PDAC patients with familial pancreatic cancer (FPC) are more likely to carry germline PVs but there is no consensus main gene involved in FPC. We performed a systematic review of publications from PubMed and Scopus reporting PVs in patients with FPC, sporadic pancreatic cancer (SPC) and unselected cohorts of PDAC patients undergoing genetic testing and calculated a cumulative prevalence of PVs for each gene evaluated across these three groups of patients. When available, variants in the selected publications were reclassified according to the American College of Medical Genetics and Genomics classification system and used for prevalence calculations if classified as pathogenic or likely pathogenic. We observed an increased prevalence of PVs in FPC compared to SPC or unselected PDAC patients for most of the 41 genes reported. The genes with the highest prevalence of carriers of PVs in FPC were *ATM, BRCA2*, and *CDKN2A*. *BRCA2 and ATM* showed the highest prevalence of PVs in both SPC and unselected PDAC cohorts. Several genes with the highest prevalence of PVs are involved in breast and ovarian cancer suggesting strong overlap with underlying genetics in these disorders but no single gene was predominant. More research is needed to further understand the risk of PDAC associated with these many diverse genes.

## Introduction

Pancreatic cancer (PC) is currently the fourth highest cause of cancer death in developed countries and pancreatic ductal adenocarcinoma (PDAC), its most common form, is associated with the worst prognosis [1]. PC is usually diagnosed at an advanced stage, which is often due to nonspecific symptoms and lack of sensitive and specific tumor markers [2]. However, recent advances in PDAC surveillance underscore the importance of identifying patients at higher risk of developing this cancer [3,4].

It has been estimated that 10% of PDAC cases have a genetic basis. Many germline pathogenic variants (PVs) in cancer predisposition genes (CPGs) that are part of several cancer predisposition syndromes (CPS) related to breast, ovarian and colorectal cancer have been identified in patients who also have PDAC. The risk of PDAC related to germline PVs in these genes is increased and varies depending on the gene involved. The highest risk is observed in patients harboring PVs in *STK11*, with a relative risk (RR) of 132 (95% CI, 44–261) while the lowest RR [2.20 (95% CI, 1.26–4.06)] is estimated for *BRCA1* PV carriers. In addition, germline PVs confer a high risk of PDAC in patients carrying PVs in other genes including *CDKN2A* (RR 13–39), the Lynch syndrome genes *MLH1*, *MSH2*, and *MSH6* (RR of 8.6–11), and *ATM*, in which a RR of 3.92 (95% CI, 0.44–14.2) has been reported [5]. Furthermore, unlike several other types of cancers, there is no single predominant gene that is most frequently involved in susceptibility for pancreatic cancer [6].

Risk of PC is also higher in patients with a positive family history and a subset of these familial pancreatic cancer (FPC) kindreds carry PVs in CPGs [7]. FPC is usually defined by kindreds that contain at least two first degree relatives with PDAC [8] usually without an identifiable syndrome in the family, whereas sporadic pancreatic cancer (SPC) is usually described as PC in an individual who has no strong family history of PDAC or another cancer, but it is also sometimes used more broadly for all individuals with PDAC who do not meet criteria for FPC.

The focus of this systematic review was to evaluate PVs identified in three distinct groups of patients with a diagnosis of PDAC not meeting criteria for a CPS and with varying degrees of familial aggregation: FPC and those not meeting criteria for FPC (non-FPC). Given the methodology of the articles reviewed, the non-FPC group was subdivided into 2 cohorts: those selected due to reported absence of family history of PDAC (SPC group) and those in which family history of PDAC was not used in the inclusion criteria for patient selection but for which it was not possible to classify as SPC and therefore this group might have had a family history of PDAC or other cancers but did not meet criteria for FPC or any other CPS (unselected PDAC group). We estimated the cumulative frequency for carriers of PVs in each gene reported and determined which genes were most frequently involved in patients with PDAC from each group. Furthermore, we conducted careful analysis and updated the classification of the specific PVs found in these PC patients, when possible.

## Materials and Methods

**Figure 1.**
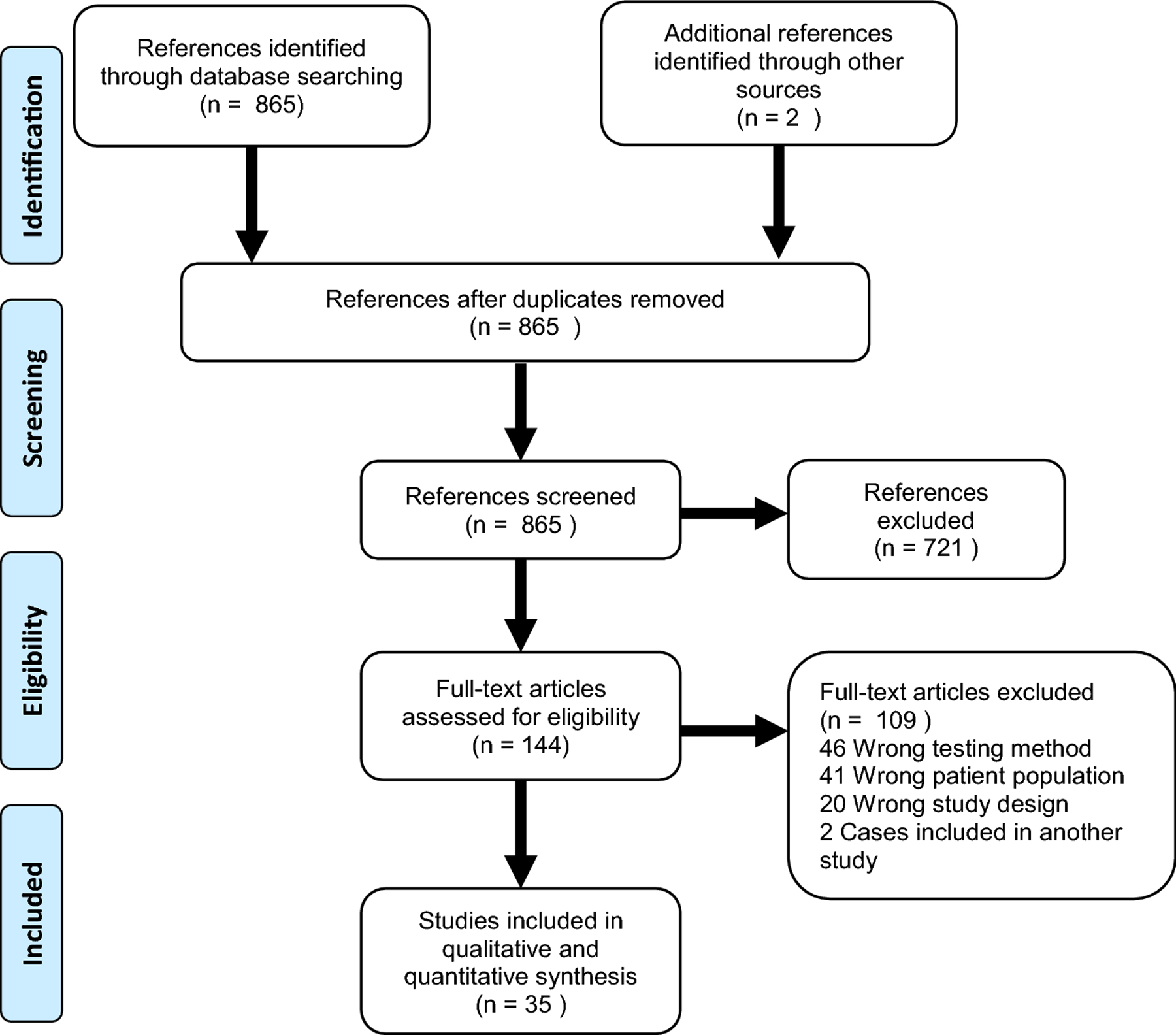
PRISMA 2009 flow diagram of included and excluded articles. A total of 865 articles were identified by database searches. Two other articles were also added after reviewing reference lists from selected articles. Two articles were excluded for being duplicates. After abstract screening of these references, 721 were determined to be irrelevant to our review because the article did not report any patients, reported somatic mutations or was related to another type of cancer or diagnosis. The full texts of a total of 144 references were then reviewed. From these, 109 references were excluded because of: 1) wrong study types (systematic and other type of reviews, meta-analyses, case reports/series, clinical trials and randomized control trials), 2) wrong patient population (diagnoses other than pancreatic ductal adenocarcinoma, or patients selected for meeting criteria for another cancer susceptibility syndrome), 3) wrong testing method (studies targeting specific variants or genotyping studies) or 4) cases reported in 2 different articles. A total of 35 articles were included for analysis and synthesis.

We followed the Preferred Reporting Items for Systematic Reviews and Meta-Analyses (PRISMA) system, including a checklist and flowchart (Figure 1). Also, Covidence online software was used for abstract screening and full text review (Covidence systematic review software, Veritas Health Innovation, Melbourne, Australia).

Germline variants associated with PDAC risk were identified through systematic literature review. Articles that identified germline variants in cohorts of patients with 1) FPC, 2) PDAC unselected for family history, and 3) SPC, were retrieved from the PubMed and Scopus databases since inception to August, 2020. The search phrase used was: ((pancreatic cancer) OR (pancreatic neoplasm)) AND (germline mutation) (((((“pancreatic neoplasms”[MeSH Terms] OR (“pancreatic”[All Fields] AND “neoplasms”[All Fields])) OR “pancreatic neoplasms”[All Fields]) OR (“pancreatic”[All Fields] AND “cancer”[All Fields])) OR “pancreatic cancer”[All Fields]) OR ((((“pancreatic neoplasms”[MeSH Terms] OR (“pancreatic”[All Fields] AND “neoplasms”[All Fields])) OR “pancreatic neoplasms”[All Fields]) OR (“pancreatic”[All Fields] AND “neoplasm”[All Fields])) OR “pancreatic neoplasm”[All Fields])) AND ((((“germ-line mutation”[MeSH Terms] OR (“germ line”[All Fields] AND “mutation”[All Fields])) OR “germ line mutation”[All Fields]) OR (“germline”[All Fields] AND “mutation”[All Fields])) OR “germline mutation”[All Fields]).

Hereditary pancreatitis genes (*PRSS1, SPINK1, CFTR, CTRC*, and *CPA1)* were not included in this review because: 1) the mechanism for development of PDAC seen with PVs in these genes may be biologically different than for other cancer predisposition genes, and 2) our search strategy was not optimal for comprehensive collection and review of articles reporting PVs in these genes.

Published articles identified based on the listed search criteria and available in any language were retrieved, imported to Covidence, screened and reviewed. During the first step, references were determined to be relevant (or not) for the review based on screening of the paper’s abstract and title. All references included after abstract screening were evaluated for risk of bias assessment using the Joanna Briggs Institute (JB) checklist (https://joannabriggs.org/critical-appraisal-tools). Questions about inclusion of an article were resolved by discussion between the authors. Full texts from relevant articles were uploaded to Covidence and reviewed according to inclusion and exclusion criteria. Articles were selected if they reported a cohort of individuals with PDAC in which genetic testing (candidate gene sequencing, whole exome sequencing or whole genome sequencing) had been done to identify germline PVs. Articles using targeted testing for specific variants (as opposed to full gene sequencing) were excluded, except for targeted *BRCA1* and *BRCA2* sequencing for patients of Ashkenazi Jewish ethnicity, which has been established to be comparable to full gene sequencing in this population [9]. Articles discussing other types of pancreatic cancer were excluded. Articles discussing copy number variants were also considered beyond the scope of this study. The diagnosis of PDAC was taken as reported by the authors.

The following information was extracted from the filtered/included articles: first author, publication year, gene name, numbers of patients tested and number of PVs identified per gene, genetic testing modality used, specific variants if available, type of pancreatic cancer cases (FPC, SPC or unselected). All variants reported by the authors as “pathogenic”, “likely pathogenic, “deleterious” or a “mutation” were collected.

Subsequently, variants collected for the included articles were reclassified using the American College of Medical Genetics and Genomics (ACMG) and the Association for Molecular Pathology’s Standards and Guidelines for the Interpretation of Sequence Variants. The detailed methods for this classification have been published [10]. Briefly, population frequency from the Genome Aggregation Database (gomAD) (https://gnomad.broadinstitute.org/), disease specific variant data from ClinVar (https://www.ncbi.nlm.nih.gov/clinvar/), and computational *in-silico* metaprediction from Franklin (https://franklin.genoox.com/clinical-db/home) were used to provide evidence of pathogenicity for each variant collected. If as a result of reclassification a variant was downgraded to variant of uncertain significance (VUS), likely benign (LB) or benign (B), the variant was not considered further for the quantitative analyses to determine the prevalence of PVs for that gene.

After collection of data for all patients carrying PVs from the included articles, we categorized patients by cohort (FPC, unselected PDAC and SPC) and gene involved. To account for potentially inflated prevalence of PVs in genes from studies with small samples, we combined all patients reported to carry a PV (numerator) and the total patients tested (denominator) across all pertinent studies to calculate a cumulative prevalence for each gene in the three separate cohorts. To avoid positive-result bias, the number of patients tested for a specific gene in every article was collected when available regardless of the result (presence vs absence of PV) and was taken into account for the total number of patients tested for that gene (denominator).

## Results

Figure 1 shows the PRISMA flowchart for this systematic review. A total of 865 articles were identified by database searches. Two other articles were also added after reviewing reference lists from selected articles. Two articles were excluded for being duplicates. After abstract screening of the 865 references, 721 articles were determined to not be relevant to the review because the title stated that the article was a review or presented management guidelines or the abstract reported somatic testing or testing of patients with a different type of cancer (i.e. not PC). The full texts of the remaining 144 references were then reviewed. From these, 109 references were excluded because of: 1) inappropriate study types (systematic and other type of reviews, meta-analyses, case reports/series), 2) erroneous patient population (diagnoses other than PDAC or patients selected for meeting criteria for another cancer susceptibility syndrome), 3) unsuitable testing method (studies targeting specific variants or genotyping studies) or 4) duplicate reporting of cases in different articles. Two pairs of articles were identified that included the same cohorts of patients; for each pair of articles, only one study was included in the review. In one situation, only one of the studies reported the specific variants found and thus this study was retained. For the second pair of studies, the most recent study was included. In total, 35 articles passed the filtering criteria and were included in the review [11–45]. No articles were excluded after risk of bias assessment using the JBI checklist. If patients in a study spanned more than one type of cohort (FPC, unselected PDAC and SPC), patients were separated and assigned to their respective cohorts for prevalence calculations. Three of the articles reported cohorts comprised of both FPC patients and patients with PDAC who had a family history of PDAC but who did not meet strict criteria for FPC (i.e. ≥2 first-degree relatives with PC). This latter group of patients from these three articles was therefore included in the unselected PDAC cohort instead of the FPC for prevalence calculations. Two articles that passed all our filtering criteria reported only absence of PVs and were also included for prevalence calculations. See Supplementary Table 1 for further details on the 35 articles included.

Overall, these 35 articles included 14,887 PDAC patients of which 1,333 were found to have PVs in one of the presented genes for a cumulative frequency of 8.95% of all patients reported and included (Supplementary Table 2). For patients with FPC, 157 carriers of PVs were identified out of a total of 1198 patients (13.1%). Frequencies of carriers of PVs in patients with unselected PDAC and SPC were 9.3% (1,133/12,177) and 2.77% (42/1,512), respectively.

Table 1 presents the genes with a cumulative frequency of PVs of at least 0.25% reported in patients with PDAC across the three groups (FPC, unselected PDAC, SPC). Full details of the 41 total genes reported are shown in Supplementary Table 2. When combining all patients from the different groups, the genes with the highest frequency of PVs reported were *BRCA2* (2.9%), *ATM* (2.52%), *CHEK2* (1.15%) and *BRCA1* (0.99%). The genes most commonly tested per number of studies were *BRCA1* (n=28), *BRCA2* (n=27) and *PALB2* (n=24). The same genes were the ones most commonly tested based on number of patients.

Among the FPC cohorts, *ATM* (3.09%) and *BRCA2* (2.61%), two genes associated with breast and ovarian cancer, had the highest prevalence of PVs followed by *CDKN2A* (2.24%), the major high-risk susceptibility gene involved in familial melanoma (Table 2). Among other genes with a prevalence of PVs >1% were *APC* (1.12%), involved in hereditary colorectal cancer, the breast cancer genes *PTEN* and *BRCA1* (1.61%, 1.06%), and *FANCA* (1.04%), involved in Fanconi anemia. The genes most frequently tested per number of studies were *PALB2* with 6 studies, followed by *ATM*, *BRCA1*, *BRCA2* and *CHEK2*, which were tested in 5 studies each. Similarly, these genes were the most commonly tested by number of patients.

Table 3 describes the genes with a frequency of PVs for unselected PDAC patients of at least 0.2%. For this group, PVs were most frequently observed in *BRCA2* (3.16%), *ATM* (2.59%) and *CHEK2* (1.26%). Other genes with a prevalence of ≥0.5% were *BRCA1*, *CDKN2A*, *MUTYH*, *PALB2* and *FANCM*. The genes most commonly tested both by number of studies and number of patients were *BRCA1* and *BRCA2*. In patients with SPC, the highest frequencies of PVs were identified in *BRCA2* (1.39%), *ATM* (1.17%) and *BRCA1* (0.33%). Six other genes (*PALB2*, *BRIP1*, *CDKN2A*, *RAD51C*, *RECQL4* and *TP53*) were also reported to carry PVs in patients from this group. When the SPC and unselected PDAC patient groups were combined to create a non-FPC cohort, the prevalences remained very similar to the unselected cohort prevalences (Supplementary Table 2).

A total of 782 PVs were collected and classified from patients for whom PVs were reported. From these variants, 472 variants were unique. Following reclassification of variants according to ACMG guidelines for variant interpretation, 29 unique variants (6.1%) were reclassified as VUS or B/LB. Given the possibility of not having complete data (*de novo* inheritance, etc.) to justify an author’s P/LP classification for some of the variants that were downgraded, we also used ClinVar to review variants before downgrading them to VUS or B/LB. Of note, none of the variants downgraded to VUS, LB or B currently have an interpretation of P/LP on ClinVar with 2 or more stars (criteria provided, multiple submitters, no conflicts).

Supplementary Table 3 shows the 11 variants each seen in more than 5 patients with PDAC. The 11 variants included 4 *ATM* variants, 2 each in *BRCA1* and *BRCA2*, and one each in *CDKN2A*, *CHEK2*, and *NBN*. Nine of the frequently observed variants are known founder PVs including 4 Ashkenazi Jewish (AJ) founder mutations in *BRCA1*, *BRCA2*, and *CHEK2*. Overall, the most frequently reported variants were the four AJ founder mutations: *BRCA2* c.5946del (also known as 6174delT), *CHEK2* c.1100del, *BRCA1* c.68_69del and *BRCA1* c.5266dup [46–48].

## Discussion

This systematic review of germline PVs in patients with a diagnosis of PDAC seeks to further elucidate the genetic complexity of this cancer and quantify the prevalence of PVs for the main genes associated with different cohorts of patients with this malignancy without a recognizable CPS.

For the patients reported in the articles included here, PVs were most frequently reported in *ATM, BRCA2* and *CDKN2A* for FPC cohorts and in *BRCA2, ATM* and *CHEK2* in patients with PDAC unselected for family history. For patients with SPC, PVs in *BRCA2*, *ATM* and *BRCA1* were the most frequently identified. With the exception of *CDKN2A*, all of the genes with the highest prevalences of PVs for each group (FPC, unselected PDAC, SPC) are involved in breast and ovarian cancer (Supplementary Figure 1), which suggests that genes associated with breast and ovarian cancer risk are also the genes most strongly associated with risk for PDAC [49].

However, the frequencies of PVs in the main genes reported in the selected articles for individuals with PDAC were remarkably similar and no particular gene was found to have PVs in more than 3.5% of the cases for any type of patient (FPC, unselected PDAC or SPC). This is in contrast to other types of cancer like hereditary breast cancer, for which *BRCA1* and *BRCA2* account for more than half of the PVs identified in patients with this diagnosis. Indeed, a recent study detected PVs in these two genes in 7.5% of patients with breast cancer unselected for family history and another 6.5% carried PVs in 19 other breast cancer genes [50].

For most of the genes reported, there was an overall higher frequency of germline PVs in cohorts of patients with FPC versus PC patients unselected for family history of cancer. There were some exceptions to this pattern, as observed for *BRCA2* and *CHEK2*, but these differences were not significant. However, there were important differences in the number of articles reporting FPC patients versus unselected PDAC, which could affect the comparability of the estimates for these two groups. Also, we cannot completely exclude the possibility that some of the articles of unselected patients might have included individuals with family history of PDAC or other cancers that was not reported or known, even if they did not meet criteria for a specific CPS. However, comparison of the prevalence of carriers of *BRCA2* PVs in FPC patients versus SPC patients showed a lower prevalence in the latter, as would be expected. No articles reported *CHEK2* PVs in SPC patients.

PVs in some genes involved in CPS previously reported to be associated with PDAC including *STK11, MSH2 MSH6, MLH1, APC* and *PMS2* in hereditary colorectal cancer, unexpectedly, had relatively low prevalences (<1%) with only *APC* reaching a cumulative frequency of >1% for any group of patients (FPC). This finding could be an artifact because of the selection criteria used for this study. Specifically, although patients with PVs in these genes may be at high risk of developing PDAC, their risks for other gastrointestinal cancers may also be high and patients may therefore present with other gastrointestinal cancers, probably earlier than with PDAC. Thus, these patients would have been excluded from cohorts of patients with only FPC, unselected PDAC or SPC. Further, it is possible that genes related to colorectal cancer, although relevant for PDAC in the context of other gastrointestinal cancers, might not play as important a role in patients and families with PDAC in the absence of family history for these other cancers. Indeed, most of the articles specifically testing genes related to gastrointestinal cancers showed an absence of PVs including *MSH2* (13 articles with negative results out of 19 articles), *MSH6* (11/19) and *STK11* (14/16).

In addition, we found that 10 out of the 35 articles reviewed reported variants previously described as deleterious but that were downgraded to VUS, B or LB classification because of insufficient pathogenicity criteria, which slightly decreased the overall prevalence of the respective genes involved for those articles and disproportionally affected genes reported in older articles that were recognized as PDAC genes many years ago. This underscores the importance of reevaluating previously reported variants as has been previously recognized by the ACMG [10].

Some other limitations of this review were that some articles did not provide a list of the variants reported and for these articles an updated classification was not possible. In these cases, the number of variants reported by the author was taken as reported. In addition, the patient population for the majority of the articles was predominantly non-Hispanic white. Of note, 4 of these studies included primarily Ashkenazi Jewish patients and another study had an Israeli cohort, which together account for the majority of the founder mutations reported. Three articles included a majority of Asian patients and one had a majority of individuals of African American background or Hispanic ethnicity (Supplementary Table 1). Due to this lack of diversity in the cohorts of the articles included, our findings might not be as relevant to other populations. Also, since our search did not include terms related to hereditary pancreatitis, we were not able to assess the genes associated with this entity.

Most of the articles included used multigene panels of well characterized high-risk CPGs as the testing modality to detect germline PVs. Even though we considered negative findings when calculating prevalence of PVs in each gene, this could have contributed to the higher prevalence observed in genes like *BRCA1* and *BRCA2*.

Further, even though genes associated with hereditary colorectal cancer had a low frequency of PVs in the patients from the selected articles, this does not reflect a weaker association between PDAC and these genes. This review was focused on patients with PDAC not meeting criteria for other CPS and thus, it is likely that patients with conditions such as Lynch syndrome or juvenile polyposis syndrome were not included because of the selection criteria for this review. Finally, some genes like *ERCC4* and *SDHA* had relatively high cumulative frequencies (0.40%, 0.25%) but were not investigated in many studies (Supplementary Table 2); thus, more work is needed to get better estimates of prevalence for the PVs seen in these genes.

Several studies are currently underway looking at the clinical implications in patients carrying germline PVs, specifically in *BRCA*-associated cancers including PDAC. Indeed, this has led to the FDA’s approval for the poly adenosine phosphate-ribose polymerase (PARP) inhibitor Olaparib for the maintenance treatment of adult patients with germline PVs in *BRCA1* and *BRCA2* and metastatic PDAC after patients treated with this drug were observed to have an increased progression-free survival, overall survival and overall response rate [51]. Although the benefits of specific therapies in PDAC patients with germline PVs in other genes are not completely understood, some studies have observed increased overall survival and overall response rate for *PALB2*-associated PDAC in patients treated with platinum-based therapies [52]. Therapeutic opportunities in PDAC patients carrying PVs in *ATM*, *ATR* or *CHEK2* are very limited, and the current experience is focused on the efficacy of oxaliplatin-based treatments, but this will probably change in the future, making genetic testing and counseling even more important for these patients [53].

Furthermore, it is in part due to these clinical implications that the latest National Comprehensive Cancer Network (NCCN) guidelines recommend that all patients with exocrine pancreatic cancer at any age undergo genetic testing and counseling at diagnosis. Indeed, the results of these tests can help determine the most effective treatment (https://www.nccn.org/professionals/physician_gls/pdf/genetics_bop.pdf).

In summary, patients with FPC have the highest prevalence of PV when compared to the other groups studied. The most frequent PVs in the FPC group occurred in *ATM, BRCA2* and *CDKN2A*. Similarly, *BRCA2* and *ATM* showed the highest prevalence of PVs in patients with PDAC from unselected cohorts (without meeting criteria for other syndromes) and SPC. Breast and ovarian cancer genes seemed to predominate as the main genes for PDAC in the groups studied, which might suggest shared mechanisms of disease in these disorders. Furthermore, despite the different degrees of family aggregation of PDAC across the 3 groups of patients studied, ranging from at least 2 affected first degree relatives with PDAC in FPC to potentially no family history in SPC, many of the genes with the highest PV frequency greatly overlapped suggesting that while the frequency of PVs increases with a higher degree of familial aggregation, the underlying genes are consistent. Overall, the six genes that had PVs with prevalences greater than one percent for any patient cohort (*ATM, BRCA1, BRCA2, CHEK2, CDKN2A and FANCA)* comprise a heterogenous group of genes that reflect the complexity of this cancer. Furthermore, no gene was predominant as seen for gene/cancer combinations such as *BRCA1* and *BRCA2* in breast and ovarian cancer, *CDKN2A* in melanoma, or *MLH1* and *MSH2* in Lynch syndrome [49, 54, 55]. Although this review focused on PVs in established PDAC genes, some articles also reported PVs in genes not traditionally associated with PDAC. However, more research is needed to further understand the risk of PDAC associated with these many diverse genes.

## Supplementary Material

1741202_Sup_tab

## Figures and Tables

**Table 1.** Genes with a cumulative frequency of pathogenic and likely pathogenic variants of at least 0.25% by patient group.

Gene	No. of studies	Frequency in FPC^a^	Frequency in unselected PDAC^a^	Frequency in SPC^a^	Cumulative frequency	No. of carriers of PVs	Total no. of patients tested
*BRCA2*	27	2.61%	3.16%	1.39%	2.90%	363	12507
*ATM*	20	3.09%	2.59%	1.17%	2.52%	231	9181
*CHEK2*	16	0.48%	1.26%	-	1.15%	80	6981
*BRCA1*	28	1.06%	1.08%	0.33%	0.99%	124	12577
*CDKN2A*	20	2.24%	0.89%	0.12%	0.98%	76	7780
*FANCM*	6	0.90%	0.50%	-	0.71%	9	1267
*FANCA*	4	1.04%	0.00%	-	0.65%	8	1232
*PALB2*	24	0.97%	0.65%	0.23%	0.65%	63	9737
*MUTYH*	12	0.22%	0.66%	-	0.51%	14	2727
*ERCC4*	3	0.94%	0.00%	0.00%	0.40%	6	1516
*MSH6*	19	0.00%	0.39%	-	0.35%	29	8395
*RAD50*	10	0.00%	0.36%	-	0.33%	8	2402
*FANCF*	4	0.31%	0.20%	-	0.26%	3	1148
*NBN*	15	0.59%	0.21%	-	0.26%	18	6828
*SDHA*	5	-	0.25%	-	0.25%	1	402
*FANCC*	8	0.31%	0.22%	-	0.25%	11	4442

a Prevalences of 0.00% are included for genes that were tested in at least 1 article but for which no pathogenic variants were identified, while “-“ indicates that the gene was not evaluated.

*PVs* pathogenic and likely pathogenic variants, *FPC* familial pancreatic cancer, *PDAC* pancreatic ductal adenocarcinoma.

**Table 2.** Genes with a frequency of pathogenic and likely pathogenic variants of at least 0.2% among patients with familial pancreatic cancer.

Gene	Number of studies	Frequency of carriers of PVs	Number of carriers of PVs	Total number of patients tested
*ATM*	5	3.09%	32	1036
*BRCA2*	5	2.61%	27	1036
*CDKN2A*	4	2.24%	22	982
*PTEN*	2	1.61%	1	62
*APC*	4	1.12%	11	982
*BRCA1*	5	1.06%	11	1036
*FANCA*	2	1.04%	8	771
*PALB2*	6	0.97%	11	1132
*ERCC4*	1	0.94%	6	638
*FANCM*	2	0.90%	6	664
*NBN*	4	0.59%	6	1010
*BRIP1*	2	0.52%	4	771
*CHEK2*	5	0.48%	5	1036
*RECQL4*	1	0.47%	3	638
*BUB1B*	2	0.45%	3	664
*FANCC*	3	0.38%	3	797
*CDH1*	3	0.35%	3	849
*FANCF*	1	0.31%	2	638
*POLD1*	1	0.31%	2	638
*MUTYH*	4	0.22%	2	903

*PVs* pathogenic and likely pathogenic variants.

**Table 3.** Genes with frequency of pathogenic and likely pathogenic variants of at least 0.2% among unselected patients with pancreatic ductal adenocarcinoma.

Gene	Number of studies	Frequency of carriers of PVs	Number of carriers of PVs	Total number of patients tested
*BRCA2*	21	3.16%	315	9959
*ATM*	15	2.59%	189	7291
*CHEK2*	12	1.26%	75	5945
*BRCA1*	22	1.08%	108	10029
*CDKN2A*	16	0.89%	53	5944
*MUTYH*	9	0.66%	12	1824
*PALB2*	19	0.65%	50	7751
*FANCM*	4	0.50%	3	603
*MSH6*	15	0.39%	29	7359
*RAD50*	8	0.36%	8	2215
*TP53*	14	0.26%	19	7189
*SDHA*	5	0.25%	1	402
*FANCC*	5	0.22%	8	3645
*BRIP1*	10	0.22%	11	5105
*MITF*	5	0.22%	3	1391
*NBN*	12	0.21%	12	5818
*ATR*	3	0.20%	1	510
*ERCC6*	3	0.20%	1	510
*FANCF*	3	0.20%	1	510

*PVs* pathogenic and likely pathogenic variants.
