# Supplementary material for: A systematic review of the prevalence of germline pathogenic variants in patients with pancreatic cancer
Source: J Gastroenterol. Author manuscript; Available in PMC 2022 Aug 1. (PMC8475496; doi:10.1007/s00535-021-01806-y)
Supplement: 1741202_Sup_tab [file NIHMS1741202-supplement-1741202_Sup_tab.docx]

**Supplementary Table 1. Articles included in systematic review.**

| **Reference** | **Country** | **Population/race/ethnicity** | **Publication source** | **Genes evaluated^a^** | **Testing modality** | **Study population** |
| --- | --- | --- | --- | --- | --- | --- |
| Alimirzaie 2018 | Iran | Middle Eastern | Arch Iran Med Jun 2018;21(6):228-233 | *APC, ATM, ATR, BARD1, BRCA1, BRCA2, BLM, CDKN2A, BRIP1, BUB1B, CDH1, CHEK2, EPCAM, ERCC4, ERCC6, FANCA, FANCC, FANCF, FANCM, FH, MITF, MEN1, MLH1, MRE11A, MSH2, MSH6, MUTYH, NBN, NF1, PALB2, PMS2, PTEN, RAD50, RAD51C, RAD51D, RECQL4, SDHA, STK11, TP53, VHL* | Multigene panel | Unselected |
| Axilbund 2009^b^ | USA | NHW (93.9%), AJ ancestry (13.6%), Hispanic (1.5%) and AA (1.5%) | Cancer Biol Ther Jan 2009;8(2):131-5 | *BRCA1* | Single gene sequencing | FPC |
| Blair 2018 | USA | NHW (84%) | J Am Coll Surg Apr 2018;226(4):630-637.e1 | *BRCA2, BRCA1* | Multigene panel | SPC |
| Borecka 2016 | Czech Republic | NHW | Cancer Genet May 2016;209(5):199-204 | *PALB2* | Single gene sequencing | Unselected |
| Brand 2018 | USA | NHW (86%), AJ ancestry (9%) | Cancer Sep 2018;124(17):3520-3527 | *ATM, APC, BRCA1, BRCA2, CDKN2A, MSH6, PALB2, PMS2, TP53, BARD1, CHEK2, NBN, NF1, POLE, POLD1, RAD51D, RAD1C, MUTYH, MRE11A, BRIP1, EPCAM, MLH2, MSH2, STK11, MLH1* | Multigene panel | Unselected |
| Chaffee 2018^c^ | USA | NHW (96.6) | Genet Med Jan 2018;20(1):119-127 | *ATM, BARD1, BRCA1, BRCA2, CDKN2A, CHEK2, MSH2, MUTYH, PALB2, PMS2, NBN, APC, EPCAM, MLH1, MSH6, STK11, TP53, BRIP1, CDH1, RAD51D* | Multigene panel | FPC, Unselected |
| Cremin 2020 | Canada | NHW (70%), Asian (21.5%), AJ ancestry (1.3%) | Cancer Med Jun 2020;9(11):4004-4013 | *ATM, APC, BRCA2, BRIP1, CDKN2A, CHEK2, MITF, MSH2, MUTYH, NBN, BARD1, BRCA1, EPCAM, MLH1, MSH6, PALB2, PMS2, POLD1, RAD51C, RAD51D, STK11, TP53, SDHA* | Multigene panel | Unselected |
| Earl 2020 | Spain | Unspecified | EBioMedicine Mar 2020;53():102675 | *CDKN2A, MLH1, FANCM, PTEN, CHEK2, APC, ATM, BRCA1, BRCA2, CDH1, EPCAM, MSH2, MSH6, MUTYH, PALB2, PMS2, STK11, TP53, VHL, FANCG, BUB1B, FANCC* | Multigene panel | FPC |
| Ferrone 2009 | Italian | AJ ancestry | J Clin Oncol Jan 2009;27(3):433-8 | *BRCA1, BRCA2* | Founder variant analysis | Unselected |
| Ghiorzo 2012 | Italian | NHW | J Med Genet Mar 2012;49(3):164-70 | *CDKN2A* | Single gene sequencing | FPC, Unselected |
| Golan 2020 | Israel | Middle Eastern | J Clin Oncol May 2020;38(13):1442-1454 | *BRCA1, BRCA2* | Founder variant analysis | Unselected |
| Grant 2015 | USA | NHW | Gastroenterology Mar 2015;148(3):556-64 | *ATM, BRCA1, BRCA2, MLH1, MSH2, MSH6, APC, CDKN2A, STK11, PALB2, PMS2, TP53* | Multigene panel | Unselected |
| Grant 2018 | Canada | Unspecified | Gastroenterology Feb 2018;154:719-722 | *APC, ATM, ATR, BARD1, BRCA1, BRCA2, BLM, CDKN2A, BRIP1, BUB1B, CDH1, CHEK2, EPCAM, ERCC6, FANCA, FANCC, FANCF, FANCM, MEN1, MLH1, MITF, MRE11A, MSH2, MSH6, MUTYH, NBN, NF1, PALB2, PMS2, RAD50, RAD51C, RAD51D, RECQL4, STK11, VHL, TP53* | Genome sequencing | Unselected |
| Holter 2015 | USA | NHW (71%) | J Clin Oncol Oct 2015;33(28):3124-9 | *BRCA1, BRCA2, PALB2, MSH2, MSH6* | Multigene panel | Unselected |
| Hu 2016 | USA | NHW (97%) | Cancer Epidemiol Biomarkers Prev Jan 2016;25(1):207-11 | *BRCA1, BRCA2, ATM, BARD1, CHEK2, NBN, MSH6, FANCM, PALB2, BRIP1, RAD51C, RAD51D, MRE11A, RAD50, MLH1, MSH2, PMS2, CDH1, TP53, PTEN, STK11* | Multigene panel | Unselected |
| Hu 2018 | USA | NHW (65.9%), AJ ancestry (10.2), AA (5.1%), Asian (2.8%), Hispanic (4%) | JCO Precis Oncol 2018;2():PO.17.00291 | *APC, ATM, BARD1, BRCA1, BRCA2, BRIP1, CDH1, CDKN2A, CHEK2, EPCAM, MEN1, MITF, MLH1, MRE11A, MSH2, MSH6, NBN, NF1, PALB2, PMS2, RAD50, TP53, VHL, STK11, MUTYH, RAD51C, RAD51D, POLD1, SDHA, FH* | Multigene panel | Unselected |
| Hu 2018 | USA | NHW (95.6%), AA (1.6%), Hispanic (1.4%) | JAMA Jun 2018;319(23):2401-2409 | *ATM, BRCA2, CHEK2, BRCA1, PALB2, CDKN2A, BARD1, BRIP1, CDH1, FANCC, MLH1, MRE11A, MSH2, MSH6, NBN, NF1, PMS2, RAD51C, RAD51D, TP53* | Exome sequencing | Unselected |
| Jones 2009 | USA | Unspecified | Science Apr 2009;324(5924):217 | *PALB2* | Single gene sequencing | FPC |
| Krepline 2020 | USA | NHW (92%), AA (4%), Hispanic (2%) | HPB (Oxford) Apr 2020;(): | *ATM, APC, CHEK2, BRCA1, BRCA2, EPCAM, FANCC, PALB2, MUTYH, CDKN2A, STK11, NBN, MSH6, MLH1. PMS2, TP53* | Multigene panel | Unselected |
| Lee 2018 | South Korea | Asian | Invest New Drugs Feb 2018;36(1):163 | *BRCA1, BRCA2* | Multigene panel | Unselected |
| Lowery 2018^d^ | USA | NHW (90%), AJ ancestry (20%), AA/Hispanic (6%) Asian (4.6%) | Journal of the National Cancer Institute 2018;110(10): | *APC, ATM, BARD1. BLM, BRCA1, BRCA2, CDKN2A, CHEK2, FH, MITF, MLH1, MSH2, MSH6, MUTYH, NBN, NF1, PALB2, PMS2, RAD50, RAD51D, RECQL4, STK11, TP53* | Multigene panel | Unselected |
| Lucas 2013 | USA | AJ ancestry | Clin Cancer Res Jul 2013;19(13):3396-403 | *BRCA1, BRCA2* | Founder variant analysis | Unselected |
| Lucas 2014 | USA | NHW (95%), AJ ancestry (80%) | Cancer Jul 2014;120(13):1960-7 | *BRCA1, BRCA2* | Founder variant analysis | Unselected |
| McWilliams 2011 | USA | NHW | Eur J Hum Genet Apr 2011;19(4):472-8 | *CDKN2A* | Single gene sequencing | Unselected |
| McWilliams 2018 | USA | NHW (81%), AA (11%), Hispanic (6%), NA (0.5%) | Cancer Epidemiol Biomarkers Prev Nov 2018;27(11):1364 | *CDKN2A* | Single gene sequencing | Unselected |
| Ohmoto 2016^b^ | Japan | Asian | Pancreas Aug 2016;45(7):1056-61 | *APC, ATM, BARD1, BRCA1, BRCA2, BRIP1, CDH1, CDKN2A, CHEK2, EPCAM, FH, MEN1, MLH1, MSH2, MSH6, MUTYH, NBN, NF1, PALB2, PMS2, PTEN, RAD50, RAD51C, STK11, TP53, VHL, SDHA* | Multigene panel | Unselected |
| Roberts 2012 | USA | Unspecified | Cancer Discov Jan 2012;2(1):41-6 | *ATM* | Single gene sequencing | FPC |
| Roberts 2016^d^ | USA | NHW (96%) | Cancer Discov Feb 2016;6(2):166-75 | *APC, ATM, ATR. BARD1, BRIP1, BRCA1, BRCA2, BUB1B, BLM, CDH1, CDKN2A, CHEK2, ERCC4, FANCA, FANCC, FANCF, FANCM, FH, MEN1, MLH1, MRE11A, MSH2, MSH6, MUTYH, NBN, NF1, PALB2, POLD1, PMS2, RECQL4, TP53, VHL, STK11* | Genome sequencing | FPC |
| Salo-Mullen 2015 | USA | AJ ancestry (62%) | Cancer Dec 2015;121(24):4382-8 | *BRCA1, BRCA2, CDKN2A, PALB2, MLH1, MSH2, MSH6, PMS2* | Multigene panel | Unselected |
| Schwartz 2019 | France | Unspecified | Clin Genet Dec 2019;96(6):579-584 | *BRCA1, BRCA2, CDKN2A, ATM, FANCA, FANCC, APC, BLM, MITF, MLH1, MSH2, NBN, NF1, PALB2, PMS2, STK11, TP53, RAD50* | Multigene panel | FPC |
| Shindo 2017^c^ | USA | NHW (89%), AA (6%) | J Clin Oncol Oct 2017;35(30):3382-3390 | *BRCA1, BRCA2, PALB2, CDKN2A, TP53, BRIP1, ATM, RAD51C, MLH1, MSH2, RECQL4, ERCC4, BUB1B, CDH1, RAD51D* | Multigene panel | SPC |
| Slater 2010 | Germany | NHW | Clin Genet Nov 2010;78(5):490-4 | *PALB2* | Single gene sequencing | FPC, unselected |
| Slavin 2018 | USA | NHW (77%) | Fam Cancer Apr 2018;17(2):235-245 | *ATM, APC, BLM, BRCA1, BRCA2, BARD1, BUB1B, CHEK2, FANCC, FANCM, MSH2, MSH6, RAD50, FANCF, ATR, RECQL4, ERCC6, MLH1, NBN, NF1, PALB2, PMS2, SDHA, TP53, VHL, RAD51C* | Multigene panel | Unselected |
| Takai 2016 | Japan | Asian | Oncotarget Nov 2016;7(45):74227-74235 | *ATM, MLH1, PALB2, BRCA2, BARD1, BRCA1, BRIP1, CHEK2, MRE11, MSH2, MSH6, MUTYH, NBN, PMS1, PMS2, PTEN, RAD50, RAD51C, STK11, TP53* | Multigene panel | FPC |
| Yurgelun 2019 | USA | NHW (76%), AA (1%), Asian (10%) | Genet Med Jan 2019;21(1):213-223 | *ATM, BRCA1, BRCA2, BRIP1, CHEK2, NBN, PALB2, RAD50, RAD51C, CDKN2A, MSH2, MSH6, TP53, APC, RAD51D, CDH1, STK11* | Multigene panel | Unselected |

^a^From genes reported to have pathogenic or likely pathogenic variants in articles included in the review.

^b^Articles reporting absence of pathogenic variants.

^c^Articles retained after exclusion of another article with duplicate reporting of cases.

^d^Articles identified by reviewing reference lists of other articles.

*FPC* familial pancreatic cancer, *SPC* sporadic pancreatic cancer, *NHW* non-Hispanic white, *AA* African American, *AJ* Ashkenazi Jewish, *NA* Native American.

**Supplementary Table 2. Frequency of pathogenic and likely pathogenic variants for each patient group and overall for all genes included in review.**

| **Gene** | **Familial Pancreatic Cancer** | | | | **Non-Familial Pancreatic Cancer** | | | | | | | | | **Total** | | | |
| --- | --- | --- | --- | --- | --- | --- | --- | --- | --- | --- | --- | --- | --- | --- | --- | --- | --- |
|  |  |  |  |  | **Unselected PDAC** | | | | **Sporadic Pancreatic Cancer** | | | | **Combined Non-FPC** |  |  |  |  |
|  | No. of articles | Frequency^a^ | No. of carriers of PVs | Total tested | No. of articles | Frequency^a^ | No. of carriers of PVs | Total tested | No. of articles | Frequency^a^ | No. of carriers of PVs | Total tested | Frequency | No. of articles^b^ | Frequency | No. of carriers of PVs | Total tested |
| ***APC*** | 4 | 1.12% | 11 | 982 | 12 | 0.03% | 1 | 3917 | 0 | - | 0 | 0 | 0.03% | 15 | 0.24% | 12 | 4899 |
| ***ATM*** | 5 | 3.09% | 32 | 1036 | 15 | 2.59% | 189 | 7291 | 1 | 1.17% | 10 | 854 | 2.44% | 20 | 2.52% | 231 | 9181 |
| ***ATR*** | 1 | 0.00% | 0 | 638 | 3 | 0.20% | 1 | 510 | 0 | - | 0 | 0 | 0.20% | 4 | 0.09% | 1 | 1148 |
| ***BARD1*** | 3 | 0.00% | 0 | 877 | 11 | 0.18% | 10 | 5539 | 0 | - | 0 | 0 | 0.18% | 13 | 0.16% | 10 | 6416 |
| ***BLM*** | 2 | 0.00% | 0 | 771 | 4 | 0.18% | 2 | 1125 | 0 | - | 0 | 0 | 0.18% | 6 | 0.11% | 2 | 1896 |
| ***BRCA1*** | 5 | 1.06% | 11 | 1036 | 22 | 1.08% | 108 | 10029 | 2 | 0.33% | 5 | 1512 | 0.98% | 28 | 0.99% | 124 | 12577 |
| ***BRCA2*** | 5 | 2.61% | 27 | 1036 | 21 | 3.16% | 315 | 9959 | 2 | 1.39% | 21 | 1512 | 2.93% | 27 | 2.90% | 363 | 12507 |
| ***BRIP1*** | 2 | 0.52% | 4 | 771 | 10 | 0.22% | 11 | 5105 | 1 | 0.12% | 1 | 854 | 0.20% | 13 | 0.24% | 16 | 6730 |
| ***BUB1B*** | 2 | 0.45% | 3 | 664 | 3 | 0.00% | 0 | 510 | 1 | 0.00% | 0 | 854 | 0.00% | 6 | 0.15% | 3 | 2028 |
| ***CDH1*** | 3 | 0.35% | 3 | 849 | 8 | 0.04% | 2 | 4773 | 1 | 0.00% | 0 | 854 | 0.04% | 12 | 0.08% | 5 | 6476 |
| ***CDKN2A*** | 4 | 2.24% | 22 | 982 | 16 | 0.89% | 53 | 5944 | 1 | 0.12% | 1 | 854 | 0.79% | 20 | 0.98% | 76 | 7780 |
| ***CHEK2*** | 5 | 0.48% | 5 | 1036 | 12 | 1.26% | 75 | 5945 | 0 | - | 0 | 0 | 1.26% | 16 | 1.15% | 80 | 6981 |
| ***EPCAM*** | 2 | 0.00% | 0 | 211 | 8 | 0.04% | 1 | 2755 | 0 | - | 0 | 0 | 0.04% | 9 | 0.03% | 1 | 2966 |
| ***ERCC4*** | 1 | 0.94% | 6 | 638 | 1 | 0.00% | 0 | 24 | 1 | 0.00% | 0 | 854 | 0.00% | 3 | 0.40% | 6 | 1516 |
| ***ERCC6*** | 0 | - | 0 | 0 | 3 | 0.20% | 1 | 510 | 0 | - | 0 | 0 | 0.20% | 3 | 0.20% | 1 | 510 |
| ***FANCA*** | 2 | 1.04% | 8 | 771 | 2 | 0.00% | 0 | 461 | 0 | - | 0 | 0 | 0.00% | 4 | 0.65% | 8 | 1232 |
| ***FANCC*** | 3 | 0.38% | 3 | 797 | 5 | 0.22% | 8 | 3645 | 0 | - | 0 | 0 | 0.22% | 8 | 0.25% | 11 | 4442 |
| ***FANCF*** | 1 | 0.31% | 2 | 638 | 3 | 0.20% | 1 | 510 | 0 | - | 0 | 0 | 0.20% | 4 | 0.26% | 3 | 1148 |
| ***FANCM*** | 2 | 0.90% | 6 | 664 | 4 | 0.50% | 3 | 603 | 0 | - | 0 | 0 | 0.50% | 6 | 0.71% | 9 | 1267 |
| ***FH*** | 1 | 0.16% | 1 | 638 | 4 | 0.11% | 1 | 927 | 0 | - | 0 | 0 | 0.11% | 5 | 0.13% | 2 | 1565 |
| ***MEN1*** | 1 | 0.00% | 0 | 638 | 4 | 0.18% | 1 | 567 | 0 | - | 0 | 0 | 0.18% | 5 | 0.08% | 1 | 1205 |
| ***MITF*** | 1 | 0.00% | 0 | 133 | 5 | 0.22% | 3 | 1391 | 0 | - | 0 | 0 | 0.22% | 6 | 0.20% | 3 | 1524 |
| ***MLH1*** | 4 | 0.00% | 0 | 1010 | 16 | 0.18% | 13 | 7170 | 1 | 0.00% | 0 | 854 | 0.16% | 20 | 0.14% | 13 | 9034 |
| ***MRE11A*** | 1 | 0.00% | 0 | 54 | 7 | 0.06% | 3 | 4621 | 0 | - | 0 | 0 | 0.06% | 8 | 0.06% | 3 | 4675 |
| ***MSH2*** | 5 | 0.10% | 1 | 1036 | 15 | 0.19% | 13 | 6845 | 1 | 0.00% | 0 | 854 | 0.17% | 19 | 0.16% | 14 | 8735 |
| ***MSH6*** | 5 | 0.00% | 0 | 1036 | 15 | 0.39% | 29 | 7359 | 0 | - | 0 | 0 | 0.39% | 19 | 0.35% | 29 | 8395 |
| ***MUTYH*** | 4 | 0.22% | 2 | 903 | 9 | 0.66% | 12 | 1824 | 0 | - | 0 | 0 | 0.66% | 12 | 0.51% | 14 | 2727 |
| ***NBN*** | 4 | 0.59% | 6 | 1010 | 12 | 0.21% | 12 | 5818 | 0 | - | 0 | 0 | 0.21% | 16 | 0.26% | 18 | 6828 |
| ***NF1*** | 2 | 0.13% | 1 | 771 | 8 | 0.12% | 6 | 5049 | 0 | - | 0 | 0 | 0.12% | 10 | 0.12% | 7 | 5820 |
| ***PALB2*** | 6 | 0.97% | 11 | 1132 | 19 | 0.65% | 50 | 7751 | 1 | 0.23% | 2 | 854 | 0.60% | 24 | 0.65% | 63 | 9737 |
| ***PMS2*** | 5 | 0.10% | 1 | 1036 | 14 | 0.13% | 9 | 6971 | 0 | - | 0 | 0 | 0.13% | 18 | 0.12% | 10 | 8007 |
| ***POLD1*** | 1 | 0.31% | 2 | 638 | 1 | 0.00% | 0 | 1078 | 0 | - | 0 | 0 | 0.00% | 4 | 0.12% | 2 | 1716 |
| ***PTEN*** | 2 | 1.61% | 1 | 62 | 4 | 0.00% | 0 | 434 | 0 | - | 0 | 0 | 0.00% | 6 | 0.20% | 1 | 496 |
| ***RAD50*** | 2 | 0.00% | 0 | 187 | 8 | 0.36% | 8 | 2215 | 0 | - | 0 | 0 | 0.36% | 10 | 0.33% | 8 | 2402 |
| ***RAD51C*** | 0 | - | 0 | 0 | 10 | 0.07% | 4 | 5991 | 1 | 0.12% | 1 | 854 | 0.07% | 11 | 0.07% | 5 | 6845 |
| ***RAD51D*** | 2 | 0.12% | 1 | 854 | 10 | 0.10% | 1 | 1039 | 0 | - | 0 | 0 | 0.10% | 12 | 0.11% | 2 | 1893 |
| ***RECQL4*** | 1 | 0.47% | 3 | 638 | 4 | 0.18% | 2 | 1125 | 1 | 0.12% | 1 | 854 | 0.15% | 6 | 0.23% | 6 | 2617 |
| ***SDHA*** | 0 | - | 0 | 0 | 5 | 0.25% | 1 | 402 | 0 | - | 0 | 0 | 0.25% | 5 | 0.25% | 1 | 402 |
| ***STK11*** | 5 | 0.00% | 0 | 1036 | 12 | 0.05% | 2 | 3781 | 0 | - | 0 | 0 | 0.05% | 16 | 0.04% | 2 | 4817 |
| ***TP53*** | 5 | 0.19% | 2 | 1036 | 14 | 0.26% | 19 | 7189 | 1 | 0.12% | 1 | 854 | 0.25% | 19 | 0.24% | 22 | 9079 |
| ***VHL*** | 2 | 0.00% | 0 | 646 | 5 | 0.15% | 1 | 662 | 0 | - | 0 | 0 | 0.15% | 7 | 0.08% | 1 | 1308 |

^a^Prevalences of 0.00% were included for genes that were tested in at least 1 article but for which no pathogenic variants were identified, while “-“ was assigned to genes not tested for that patient group.

^b^Some articles included more than 1 group of patients within the same article.

*PV* pathogenic and likely pathogenic variants, *PDAC* pancreatic ductal adenocarcinoma.

**Supplementary Table 3. Pathogenic variants reported in more than 5 patients in the articles reviewed.**

| **Gene** | **Variant** | **Number of reports** | **Other names** | **Comments** |
| --- | --- | --- | --- | --- |
|  |  |  |  |  |
| *ATM* | NM_000051.3:c.1564_1565del | 6 | - | 16 ClinVar submissions, not known to be founder mutation |
| *ATM* | NM_000051.3:c.3245_3247delinsTGAT | 8 | 3245ATC>TGAT | 11 ClinVar submissions, Norwegian founder mutation |
| *ATM* | NM_000051.3:c.3802del | 7 | - | 11 ClinVar submissions,  British Isles founder mutation |
| *ATM* | NM_000051.3:c.7630-2A>C | 7 | IVS51-2A>C | 14 ClinVar submissions,  Polish founder mutation |
| *BRCA1* | NM_007294.3:c.68_69del | 18 | 185_186delAG 187delAG 185delAG  66_67del | 46 ClinVar submissions, Ashkenazi Jewish founder mutation |
| *BRCA1* | NM_007294.3:c.5266dup | 15 | 5382_5383insC p.Gln1756ProfsX74 5382insC 5384insC 5385insC 5383insC | 53 ClinVar submissions, Ashkenazi Jewish founder mutation |
| *BRCA2* | NM_000059.3:c.5946del | 59 | 6174delT | 46 ClinVar submissions, Ashkenazi Jewish founder mutation |
| *BRCA2* | NM_000059.3:c.7069_7070del | 6 | 2357delCT 7297_7298delCT 7297delCT | 26 ClinVar submissions, not known to be founder mutation |
| *CDKN2A* | NM_000077.4:c.301G>T | 9 | p.G101W:GGG>TGG | 14 ClinVar submissions,  Mediterranean founder mutation |
| *CHEK2* | NM_007194.4:c.1100del | 25 | - | 36 ClinVar submissions, Ashkenazi Jewish founder mutation |
| *NBN* | NM_002485.4:c.657_661del | 7 | 657del5 | 24 ClinVar submissions,  Slavic founder mutation |

**Supplementary Figure 1. Genes with pathogenic and likely pathogenic variants in pancreatic cancer patients from the articles included in systematic literature review classified by their association with other types of cancer.**


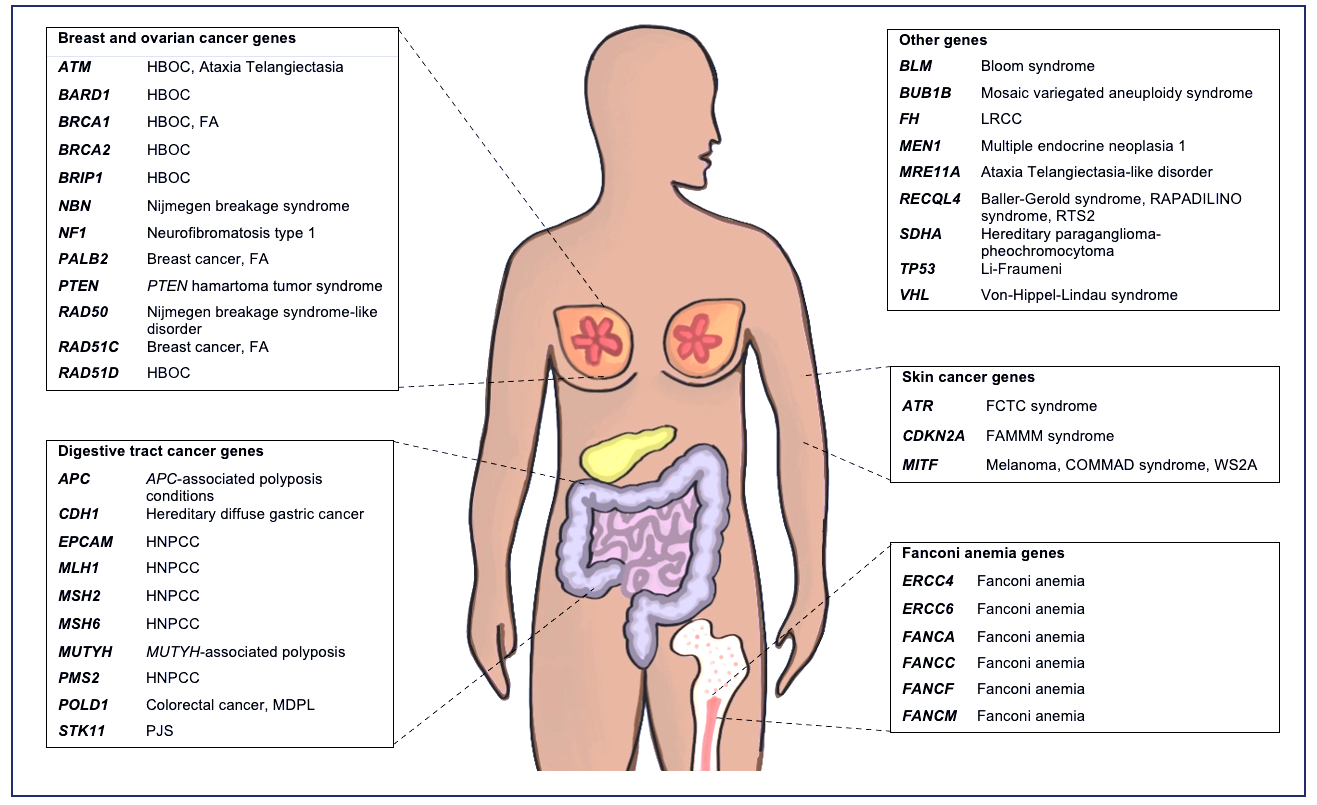

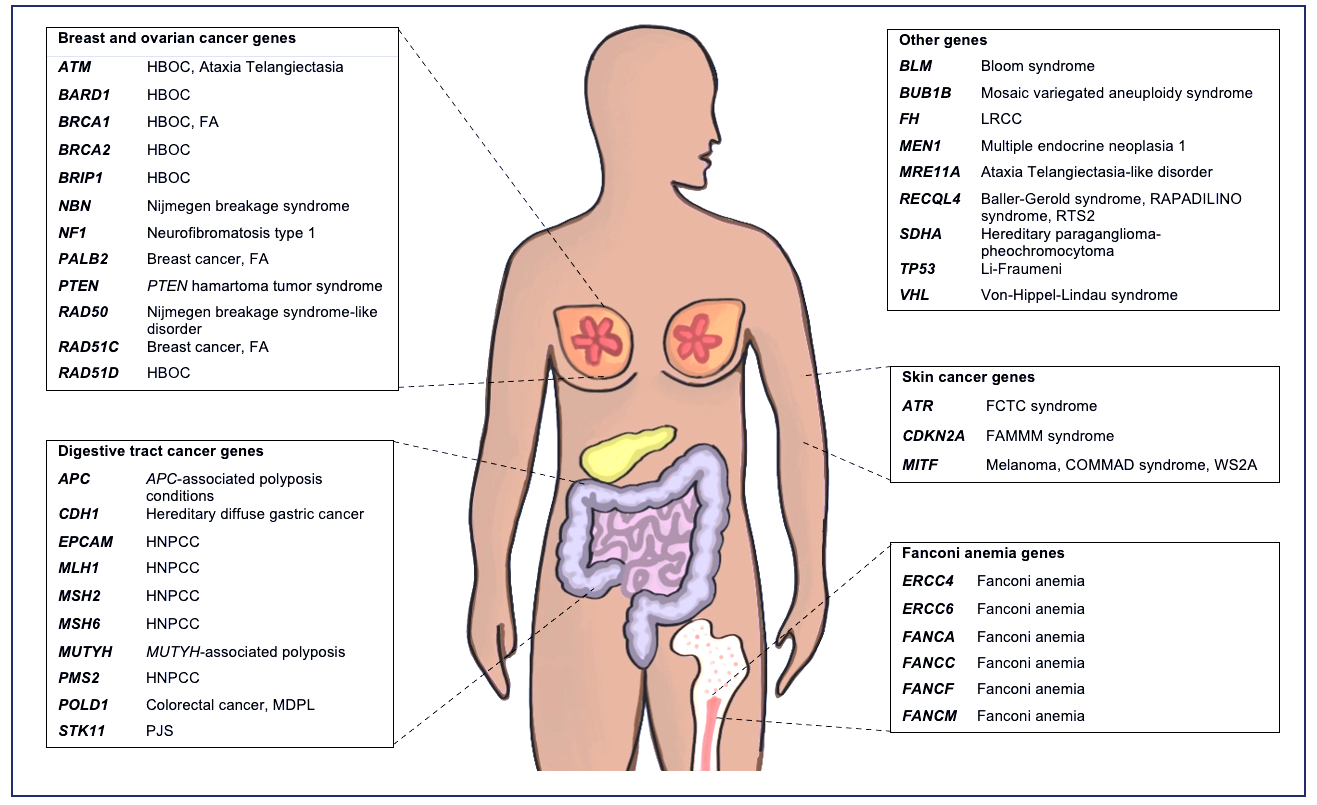

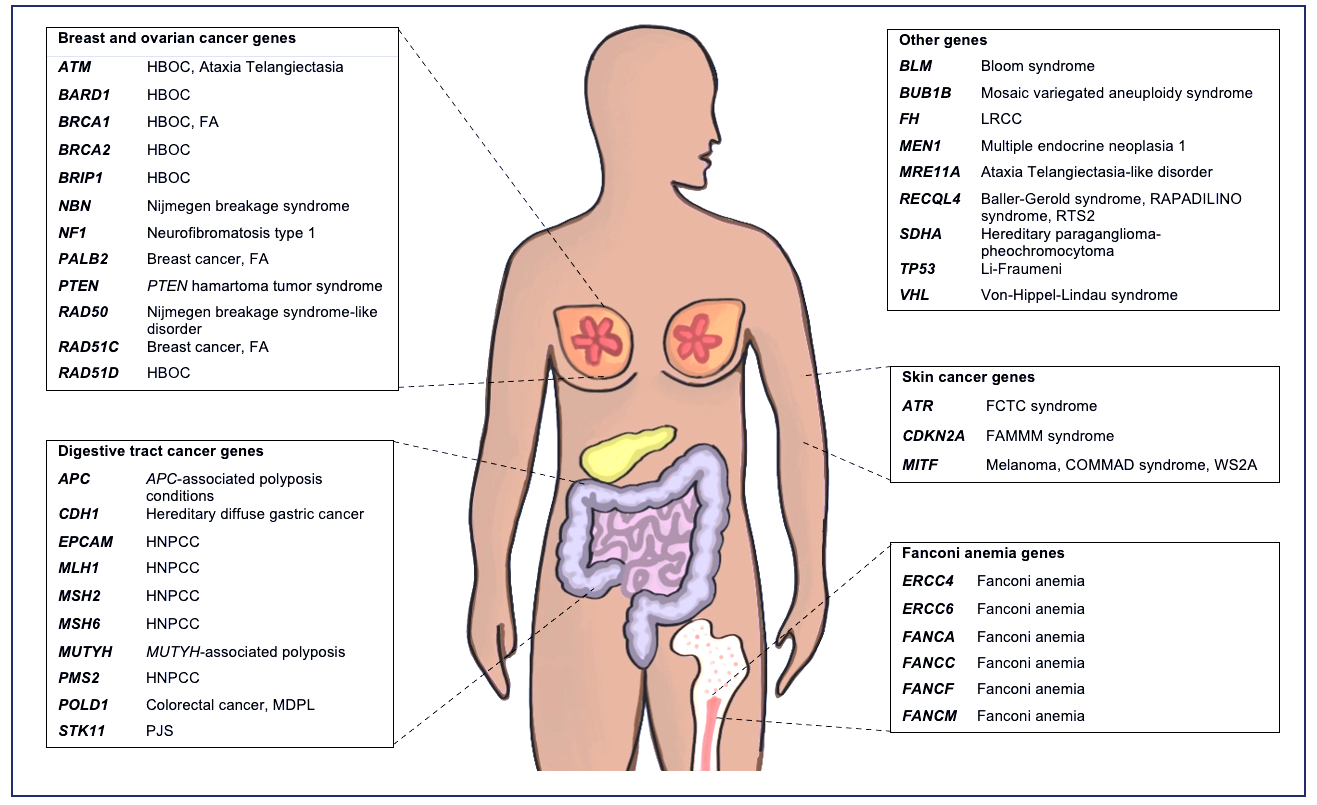


*HBOC* hereditary breast and ovarian cancer, *FA* Fanconi anemia, *HNPCC* hereditary non-polyposis colorectal cancer, *MDPL* mandibular hypoplasia, deafness, progeroid features, and lipodystrophy syndrome, *PJS* Peutz-Jeghers syndrome, *FCTC* familial cutaneous telangiectasia and cancer syndrome, *FAMMM* familial atypical multiple mole melanoma, *COMMAD* coloboma, osteopetrosis, microphthalmia, macrocephaly, albinism and deafness, *WS2A* Waardenburg syndrome 2A, *LRCC* leiomyomatosis and renal cell cancer, *RTS2* Rothmund-Thomson syndrome 2.
